# The assessment of surgical and non-surgical treatment of stage II medication-related osteonecrosis of the jaw

**DOI:** 10.4317/medoral.22013

**Published:** 2017-10-21

**Authors:** Takanori Eguchi, Ikuyo Kanai, Akihiko Basugi, Yukinaga Miyata, Minako Inoue, Yoshiki Hamada

**Affiliations:** 1 Department of Oral and Maxillofacial Surgery, Toshiba Rinkan Hospital, Sagamihara, Japan; 2 Department of Oral and Maxillofacial Surgery, School of Dental Medicine, Tsurumi University, Yokohama, Japan

## Abstract

**Background:**

Non-surgical treatment has generally been recommended for stage II medication-related osteonecrosis of the jaw (MRONJ) in preference to surgery. However, non-surgical treatment is not empirically effective. The aim of this study was to evaluate whether surgical or non-surgical treatment leads to better outcomes for stage II MRONJ.

**Material and Methods:**

In this retrospective study, surgery was performed in a total of 28 patients while 24 patients underwent non-surgical treatment. The outcomes of both treatment approaches after 6 months were evaluated and statistically compared. In addition, risk factors for surgical and non-surgical treatments were assessed for each.

**Results:**

Surgical treatment in 25 patients (89.3%) resulted in success, with failure in 3 patients (10.7%). Non-surgical treatment was successful for 8 patients (33.3%) and failed in 16 patients (66.7%). There was therefore a significant difference between surgical and non-surgical treatment outcomes (*P*<0.01). Regarding risk factors, in non-surgical treatment primary diseases, medications, and drug holiday had a significant effect on outcomes (*P*<0.01). Risk factors for surgical treatment could not be clarified.

**Conclusions:**

Surgical treatment is more effective than non-surgical treatment for stage II MRONJ, and drug holiday, primary disease, and medication constitute risk factors in non-surgical treatment.

** Key words:**Bisphosphonate, bisphosphonate-related osteonecrosis of the jaw, denosumab, management, medication-related osteonecrosis of the jaw.

## Introduction

Bisphosphonates (BPs) and denosumab are medications widely used to manage cancer-related conditions, including hypercalcemia; skeletal-related events associated with bone metastasis in the context of solid tumours such as breast, prostate, and lung cancer; lytic lesions in the setting of multiple myeloma; and osteoporosis, osteopenia, and Paget disease (1-7). However, these agents have also been reported to cause osteonecrosis of the jaw, commonly referred to as medication-related osteonecrosis of the jaw (MRONJ) (8).

MRONJ is an intractable progressive disease thought to be caused by dentoalveolar surgery, such as tooth extraction, periodontal surgery, and dental implant placement, as well as ill-fitting dentures (9-11), although around 25% of MRONJ develop spontaneously (12). Frequency of MRONJ has been reported as 0.004% to 0.1% among patients administered oral BPs, 0.017% to 6.7% for intravenous BPs, and 0.04% to 1.9% for denosumab (8). Although relatively rare, MRONJ has potentially severe symptoms in many cases. Clinical presentation is usually gingival ulceration with necrotic bone exposure in the oral cavity. As the disease progresses it leads to spontaneous severe pain, purulent drainage, extraoral fistula, and pathological fracture (13), with consequent significant reduction in quality of life.

The management of MRONJ was advocated by a staging system published in an American Association of Oral and Maxillofacial Surgeons (AAOMS) position paper in 2014, which updated the AAOMS position papers of 2007 and 2009 (8) and divides MRONJ into four stages based on clinical and radiographic findings. This system, widely used for definitive diagnosis and management of MRONJ, recommends non-surgical treatment except for stage III, because dentoalveolar surgery for MRONJ is considered hazardous and most MRONJ patients have advanced malignant disease. However, particularly for stage II patients, non-surgical treatment cannot improve the symptoms empirically. Thus, suitable management for patients with stage II MRONJ is controversial.

The aim of this study was to compare the therapeutic effects of surgical and non-surgical treatment, and to evaluate which mode of management is more effective for patients with stage II MRONJ.

## Material and Methods

- Patients

This retrospective study investigated a total of 52 patients suffering from stage II MRONJ who were referred to the Department of Oral and Maxillofacial Surgery, Toshiba Rinkan Hospital, from April 2010 to April 2015. The classification of clinical staging was based on clinical and radiographic features and according to the 2014 AAOMS position paper for MRONJ. All patients initially underwent non-surgical treatment for at least 1 month, including antibacterial mouth rinse, local irrigation, administration of antibiotics and analgesics, and professional management of oral hygiene by a dental hygienist. After evaluation at 1 month post initiation of non-surgical treatment, 28 of the 52 patients underwent surgical treatment and 24 patients continued with non-surgical treatment, because the initial treatment led to improvement of symptoms in 18 patients while the remaining six refused surgical intervention.

Clinical characteristics including age, gender (male or female), primary diseases (malignant or non-malignant), medications (intravenous BP and/or denosumab or intraoral BP), localization (maxilla or mandible), drug holidays (0-3 months or >3 months), and systemic factors (use of corticosteroids, use of tobacco, diabetes, and anaemia) were compiled, and no significant differences were found between the surgical and non-surgical treatment groups with regard to each clinical characteristic ( Table 1).

**Table 1:**
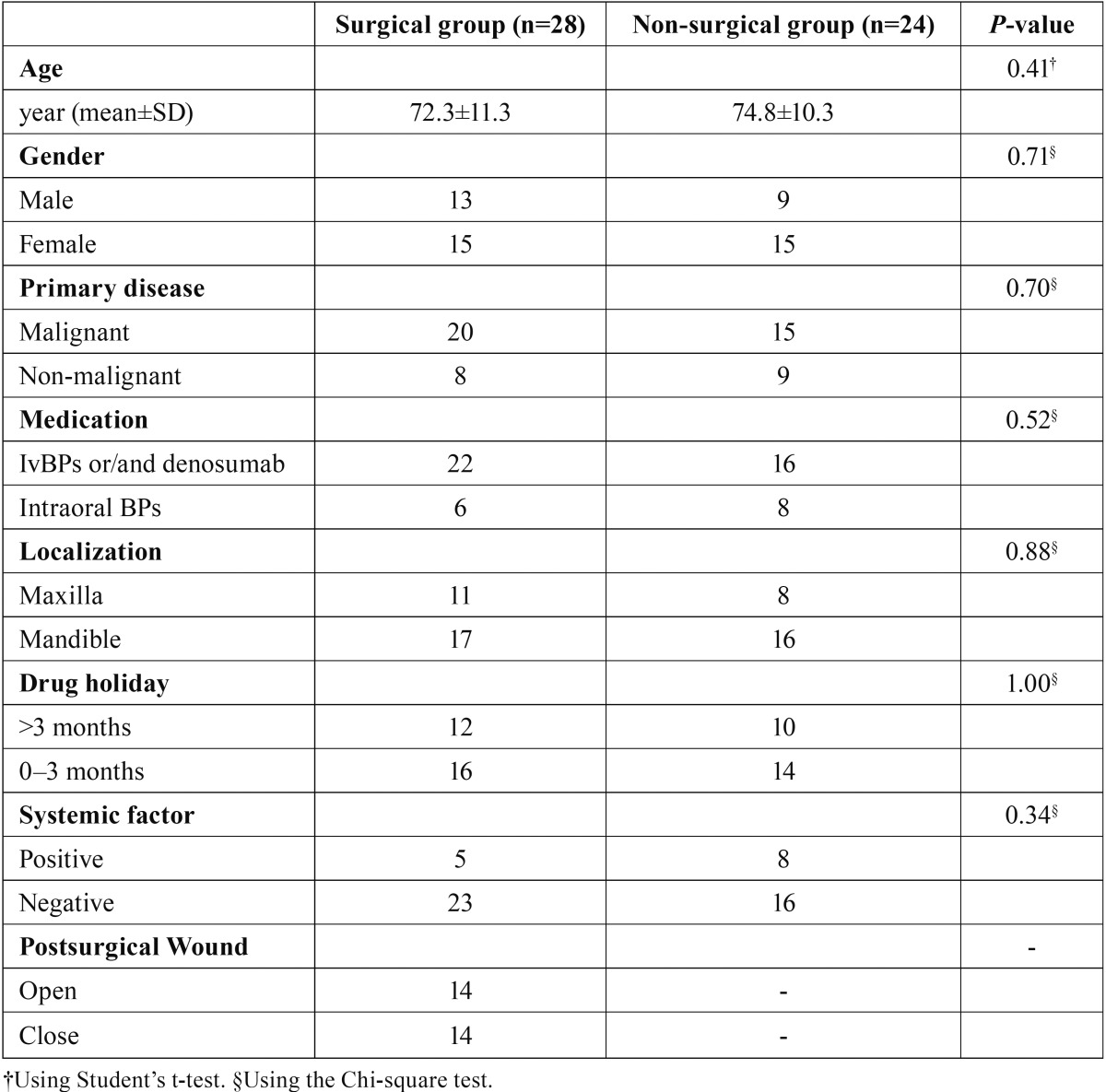
Clinical characteristics of surgical and non-surgical treatment groups. There was no significant difference between clinical characteristics of surgical and non-surgical groups.

- Clinical strategy

If it was possible to suspend BP and/or denosumab upon consultation with the treating physician, we performed surgical or non-surgical treatment with a drug holiday.

- Non-surgical treatment

Initial non-surgical treatment was continued as long as possible. Debridement to relieve soft tissue irritation and remove free necrotic bone fragments was also performed. When swelling and pain were exacerbated, we administered effective antibiotics (amoxicillin, ceftriaxone, clindamycin, and/or cefcapene pivoxil) and analgesics (loxoprofen, acetaminophen, and/or tramadol hydrochloride) accompanied by incisional drainage. These patients also underwent strict follow-up at least every 2 weeks.

- Surgical treatment

**Figure 1:**
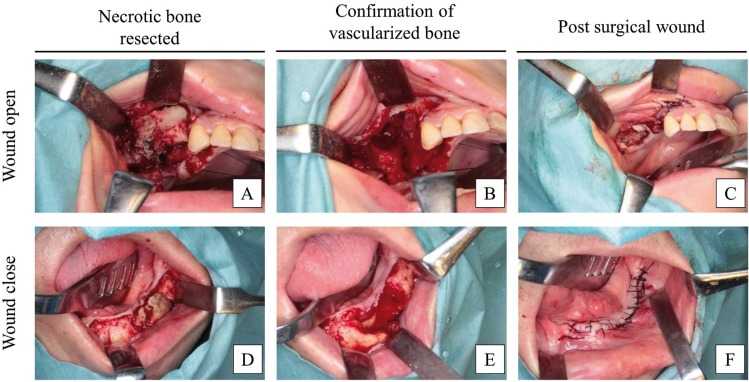
Surgical treatment process. (A) Necrotic bone of right maxilla was resected. (B) Vascularization of bone tissue was confirmed. (C) Incomplete closure of the wound and insertion of terramycin-ointment gauze. (D) Necrotic bone of left mandible was resected. (E) Vascularization of bone tissue was confirmed. (F) The wound was completely closed.

Prior to the surgical treatment, we re-evaluated the size of lesions based on radiographic examinations, including panoramic radiography, computed tomography, and magnetic resonance imaging (MRI). The surgical approach was planned according to the radiographic features. Surgery consisted of necrotic bone resection until vascularization of bone tissue was confirmed. No cases required reconstructive surgery or bone grafting. All of the resected bones were histopathologically examined to exclude other diseases such as malignant tumours. Essentially, the surgical wound was completely sutured. If complete closure of the surgical wound was impossible, the open wound was treated by inserting terramycinointment gauze (Fig. 1). Antibiotics were administered for at least 5 days after surgery, and strict follow-up was conducted at least every 2 weeks.

- Evaluation of treatment outcome

**Figure 2:**
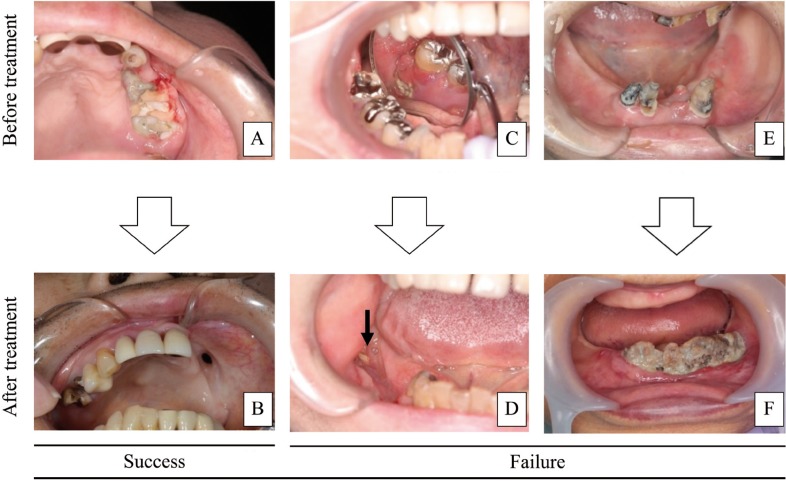
Evaluation of treatment outcome. (A) The necrotic bone exposure and pus were observed in left maxilla. (B) Disappearance of exposed bone and pus. (C) Bone exposure observed in right mandible. (D) Bone exposure remained after surgical treatment (indicated by arrow). (E) Infection and necrotic bone in left mandible. (F) Bone exposure and infection spread to right mandible.

The outcomes of both surgical and non-surgical treatments were evaluated at 6 months’ follow-up. We defined ‘Success’ as the complete disappearance of exposed bone without clinical symptoms, and ‘Failure’ as bone exposure remaining or disease progress (Fig. 2).

- Evaluation of risk factors of outcomes

To explore the risk factors for treatment outcomes, we analysed the relationship between treatment outcomes and clinical characteristics. The clinical characteristics of the non-surgical treatment group included age, gender, primary disease, medication, localization, drug holiday, and systemic factors. In addition, postsurgical wound (open or closed) was included for the surgical treatment group. Treatment outcomes were subdivided into success groups and failure groups.

- Statistical analysis

Continuous data are described as mean ± standard deviation (SD) and were analysed using Student’s t-test. Categorical data were analysed using the Chi-square test and Fisher’s exact test (less than n=5). *P* values of less than 0.01 were considered to be statistically significant. All statistical analyses were performed with EZR (Saitama Medical Center, Jichi Medical University, Saitama, Japan), which is a graphical user interface for R (The R Foundation for Statistical Computing, Vienna, Austria). More precisely, it is a modified version of R commander designed to add statistical functions frequently used in biostatistics.

## Results

- Evaluation of treatment outcomes between surgical and non-surgical groups

In the surgical treatment group, we evaluated 25 of 28 patients (89.3%) as ‘Success’ and 3 (10.7%) as ‘Failure’. The three ‘Failure’ patients had no lesional expansion and progress stage. In the non-surgical group, 8 of 24 patients (33.3%) were evaluated as ‘Success’ and 16 (66.7%) as ‘Failure’. Five ‘Failure’ patients had disease progress. There was a statistically significant difference between surgical and non-surgical treatment outcomes (*P*<0.01). An overview is provided in  Table 2.

**Table 2:**
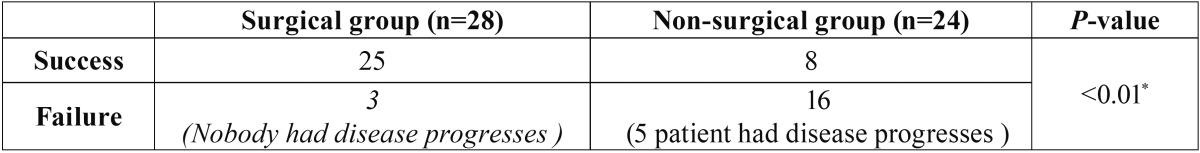
Evaluation of treatment outcomes between surgical and non-surgical groups. There was a statistically significant difference between surgical and non-surgical treatment outcomes.

- Evaluation of risk factors of outcomes in the non-surgical treatment group

The success group of non-surgical treatment comprised age (mean 80.35±11.35), gender (male/female 4:4), primary disease (malignant/non-malignant 1:7), medication (intravenous BP and/or denosumab/intraoral BP 2:6), localization (maxilla/mandible 2:6), drug holidays (>3 months/0–3 months 8:0) and systemic factors (positive/negative 3:5). On the other hand, the failure group comprised age (mean 72.00±11.02), gender (male/female 5:11), primary disease (malignant/non-malignant 14:2), medication (intravenous BP and/or denosumab/intraoral BP 14:2), localization (maxilla/mandible 6:10), drug holidays (>3 months/0–3 months 2:14) and systemic factors (positive/negative 5:11). Primary disease, medication, and drug holiday showed a significant difference between the success group and failure group (*P*<0.01). An overview is provided in  Table 3.

**Table 3:**
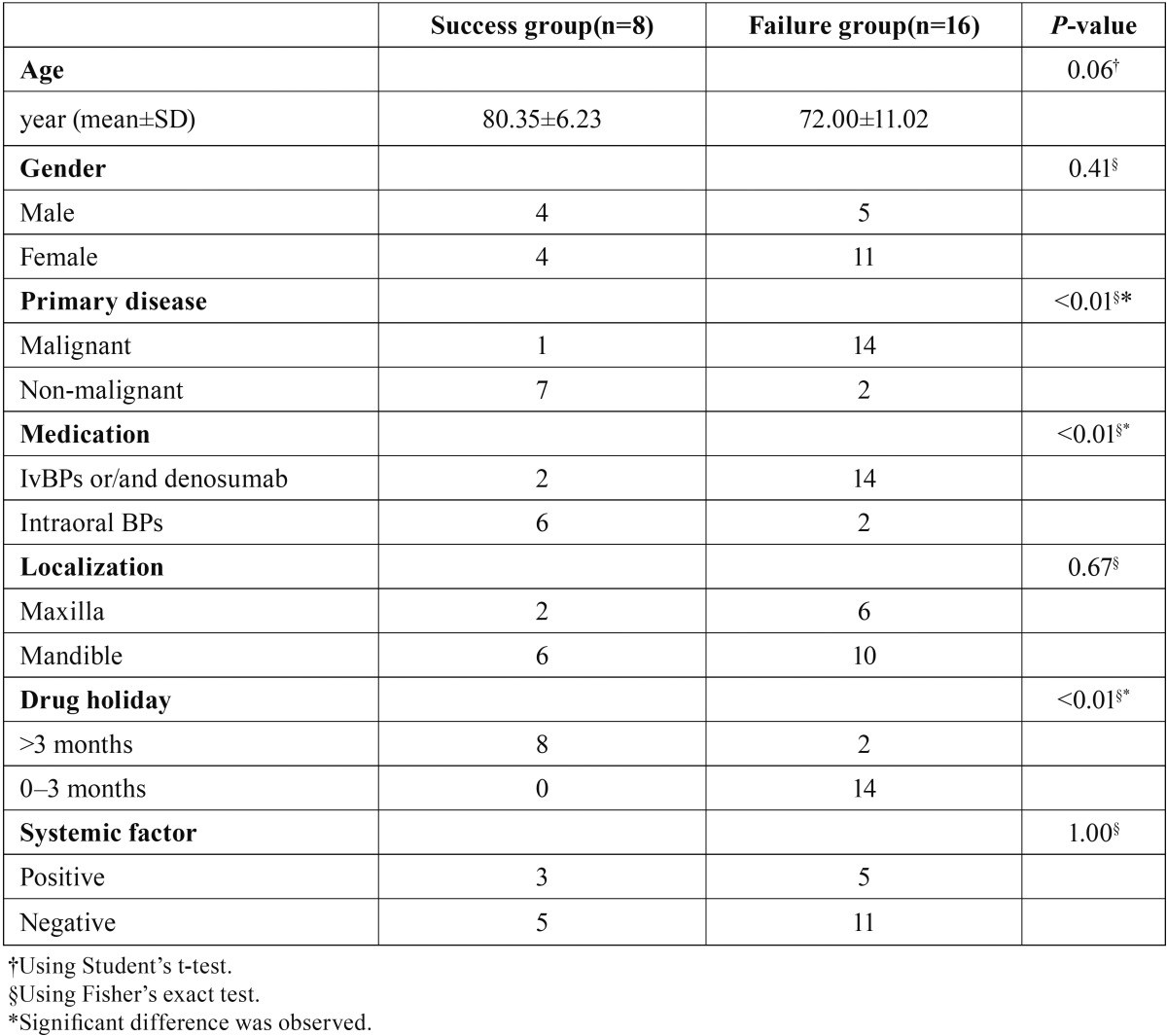
Evaluation of risk factors of outcomes in the non-surgical treatment group. Primary disease, medication and drug holiday showed a significant difference between the success group and the failure group.

- Evaluation of risk factors of outcomes in the surgical treatment group

The success group of surgical treatment comprised age (mean 73.20±11.35), gender (male/female 13:12), primary disease (malignant/non-malignant 17:8), medication (intravenous BP and/or denosumab/intraoral BP 19:6), localization (maxilla/mandible 11:14), drug holidays (>3 months/0–3 months 12:13), systemic factors (positive/negative 5:20) and postsurgical wound (open/closed 12:13). The failure group comprised age (mean 64.33±11.35), gender (male/female 1:2), primary disease (malig-nant/non-malignant 3:0), medication (intravenous BP and/or denosumab/intraoral BP 3:0), localization (maxilla/mandible 0:3), drug holiday (>3 months/0–3 months 0:3), systemic factors (positive/negative 0:3) and postsurgical wound (open/closed 2:1).

There was no significant difference between treatment outcomes and clinical characteristics in the surgical group. An overview is provided in  Table 4.

**Table 4:**
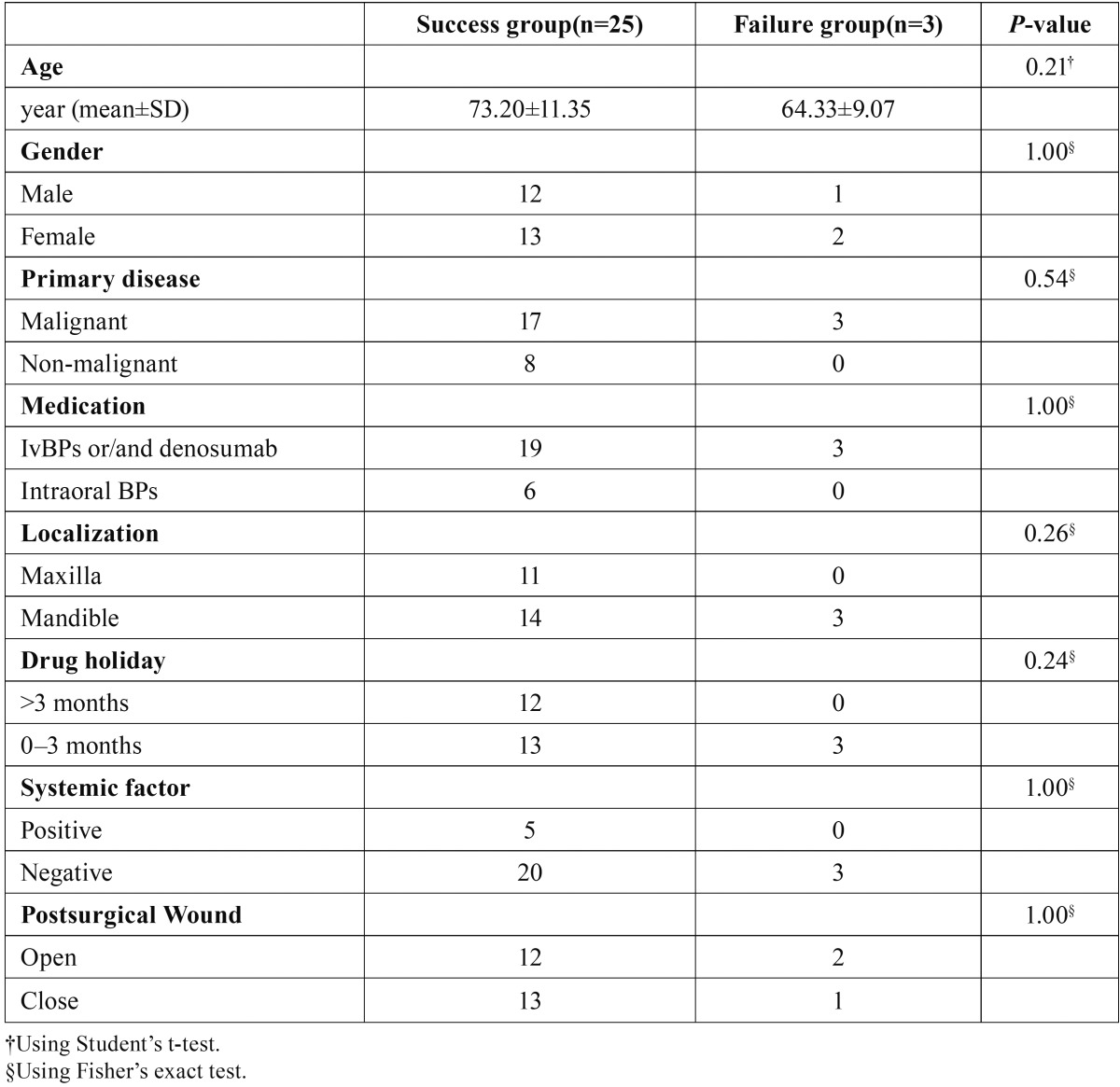
Evaluation of risk factors of outcomes in the surgical treatment group. There was no significant difference in clinical characteristics between the success group and the failure group.

## Discussion

This study revealed that surgical treatment for stage II MRONJ led to more effective improvement in comparison with non-surgical treatment (success of surgical treatment versus non-surgical treatment: 89.3% versus 33.3%). In stage II MRONJ management, the suitability of surgical or non-surgical treatment is unclear and controversial. The more common recent approach has been non-surgical treatment, and the surgical approach is rare because dentoalveolar surgery is considered to be related to the progression of MRONJ (11). However, non-surgical treatment is unable to achieve effective results empirically, with some authors reporting a success rate (complete healing) of non-surgical treatment of 17% to 23% (14–16). Meanwhile, some studies have reported a success rate of 85% to 100% for surgical treatment in recent years (17–20). We considered that surgical treatment is more suitable than non-surgical treatment for stage II MRONJ.

Many MRONJ patients have been treated according to the staging guidelines in the AAOMS position paper, where surgical intervention was recommended to treat only stage III. Stage III comprises severe clinical features such as pathological fracture, oroantral communication, severe pain, extraoral fistula, and necrotic bone extending beyond the region including ramus, sinus and zygoma. Thus, treatment of stage III has involved not only marginal bone resection and removal of necrotic bone but also segmental resection and immediate reconstruction with vascularized bone block grafting (21–23). However, performing such an expansive operation is not always suitable for patients with malignant disease. Given their poor systemic condition, bone grafting might lead to malignant disease metastasis. In general, although few patients with stage II MRONJ require reconstructive surgery, we consider that surgical treatment should be viewed positively for stage II MRONJ.

Dentoalveolar surgery has not been recommended for patients taking BPs, as many clinicians have reported MRONJ induced by tooth extraction. In fact, many cases of BRONJ were diagnosed after tooth extractions. In the present study there was no disease progress caused by surgical treatment. Sven *et al.* reported patients taking BP treatment for local infection control who underwent tooth extraction without a drug holiday, with a good result (24). Tooth extraction is indicated mainly for severe periodontitis and refractory periapical lesions. The developing MRONJ may therefore be involved with local infection control rather than the surgical intervention.

Before dentoalveolar surgery, a drug holiday has been generally recommended. However, we are particularly sceptical about the requirement for a drug holiday. In fact, our results indicated that there was no significant difference in this regard. There is currently no evidence that the length of drug holiday and interruption of anti-resorptive therapy alter the risk of osteonecrosis of the jaw (8,25,26). Specifically, a drug holiday for a patient with malignant disease may result in various adverse events; therefore, more clear evidence is necessary regarding this issue.

Although some authors have reported performing surgical treatment for MRONJ, the key to surgical success is yet to be revealed. Many studies recommend postsurgical wound closure in order to achieve treatment success. However, our present results show no significant difference in outcome whether the wound was closed or open. On examining the literature reporting satisfactory surgical results, we found that confirming vascularized bone tissue on all resected bone surfaces is an important criterion (20,24,27,28). In stage II MRONJ, it is difficult to determine the extent of the osteonecrosis only by radiographic examination or MRI, because sequestrum separation is not found. In this study, we carefully controlled the extent of resection until confirming vascularized bone tissue in the resected bone surfaces, which led to a satisfactory treatment outcome. We consider that the post-surgical wound had no influence on the outcome, and that confirming bleeding from the resection site, namely reliable elimination of necrotic bone, may hold the key to surgical success in the treatment of stage II MRONJ.

Our study indicated primary disease, medication, and drug holiday as risk factors for non-surgical treatment outcome, with the high-risk groups being patients with malignant disease, intravenous BPs and/or denosumab, and a 0- to 3-month drug holiday. This result has to be considered carefully because there are confounders involved; for example, a patient with malignant disease being administered intravenous BPs would be unable to take a longer-term drug holiday. We hypothesized that the true risk factor in non-surgical treatment is malignant disease. In support of this, there have been no reported cases of BRONJ in children or adolescents on intravenous BPs (29). In addition, the US Food and Drug Administration reported that there was no significant difference in BRONJ incidence between intravenous BPs and oral BPs in the treatment of osteoporosis. These reports suggest that intravenous BP is unlikely to be a factor that worsens or induces MRONJ, and that it may not constitute a risk for non-surgical treatment. Meanwhile, as already noted, we must remain sceptical of the influence a drug holiday may have on MRONJ incidence, owing to the lack of evidence. In patients with malignant disease, bone metabolic function becomes abnormal and resistance to infection is lowered because of malnutrition and various complications. Although further investigation is necessary, we consider that non-surgical treatment may be particularly ineffective in patients with malignant disease.

Although we used the AAMOS position paper because of its widespread adoption, we consider that this classification is not always suitable, especially in relation to treatment. AAMOS classified by clinical findings such as presence of infection, lesional expansion, and symptoms, all of course very important for classifying MRONJ. However, we feel that AAMOS position paper does not adequately consider the relationship to treatment efficacy. In fact based on the treatment strategy in AAMOS position paper, especially in stage II, treatment (non-surgical) frequently failed in our result. Bagan *et al.* classify by considering the treatment efficacy of stage 2 (uncontrolled or controlled by medication) (30). We agree with this method of classification, because this classification include treatment strategy. The classification should contribute to decision making as regards treatment methods. If the ‘Guidelines for MRONJ’ are to be further constituted, the classification has to consider treatment efficacy.
